# Characterization of the small RNAs carried by outer membrane vesicles produced by *hlyF*-positive Shiga toxin-producing *Escherichia coli*

**DOI:** 10.3389/fcimb.2025.1621341

**Published:** 2025-08-08

**Authors:** Giorgia Barbieri, Alessia Stefano, Agostina Pietrantoni, Federica Fratini, Serena Cavallero, Rosangela Tozzoli, Paola Chiani, Arianna Boni, Stefano Morabito, Valeria Michelacci

**Affiliations:** ^1^ Department of Food Safety, Nutrition and Veterinary Public Health, Istituto Superiore di Sanità, Rome, Italy; ^2^ Department of Public Health and Infectious Diseases, Sapienza University of Rome, Rome, Italy; ^3^ Istituto Superiore di Sanità, Core Facilities Technical-Scientific Service, Rome, Italy

**Keywords:** outer membrane vesicles, OMVs, small RNA, Shiga toxin-producing *Escherichia coli*, extraintestinal pathogenic *Escherichia coli*, infection, microbiota, hybrid strains

## Abstract

Shiga toxin (Stx)–producing *Escherichia coli* (STEC) harboring virulence determinants of Extraintestinal pathogenic *E. coli* (ExPEC) are currently emerging in Europe as a cause of severe disease. Among the ExPEC features identified in STEC, the gene *hlyF* is associated with an augmented production of Outer Membrane Vesicles (OMV). OMVs produced by *E. coli* strains have been shown to deliver toxins and small RNAs, but information on the latter genetic component is still scanty. We investigated the small RNAs contained in the OMVs produced by two *hlyF*-positive STEC strains producing Stx2 belonging to O26:H11 and O80:H2 serotypes, isolated from a human case of Hemolytic Uraemic Syndrome (HUS) and from a milk sample, respectively. The OMVs were purified from overnight cultures using two sequential ultracentrifugation steps at 100.000 and 200.000 x *g* of 2h each. The OMVs collected were analyzed by Nanoparticle Tracking Analysis (NTA) and Electron Microscopy (EM) to define their morphology, size and concentration. The whole genome sequences of the two test strains and three additional *hlyF*-positive STEC strains were analyzed to identify sequences of small RNAs. Specific Real Time PCR assays were deployed to detect the small RNAs within OMVs. The EM and NTA characterization confirmed the presence of spherical particles, and showed that a cleaner preparation was obtained after the second step of ultracentrifugation (200.000 x *g*). We identified the sequences of 27 putative small RNAs present in the genome of *hlyF*-positive STEC strains and absent in that of a non-pathogenic *E. coli*. Nine of them were identified and quantified in the OMVs produced by the two test strains. A regulatory role in bacterial DNA replication, integration and in stress-response was hypothesized for the identified small RNAs, some of which encompassing all the domains of life. STnc_100 was the only small RNA identified in the OMVs produced by both strains. Interestingly, a copy of STnc_100 sequence is harbored downstream the *stx2* operon carried by Stx2-encoding phages in both the strains and has a complementarity region for *stxB* gene, suggesting a modulation in Stx production or release. Our results indicate that OMVs release may exert regulatory functions, putatively influencing the crosstalk with the host and the gut microbiota during the infection process.

## Introduction

Shiga toxin-producing *Escherichia coli* (STEC) are zoonotic pathogens responsible for gastrointestinal infections in humans which can lead to serious complications, including hemorrhagic colitis (HC) and Hemolytic Uremic Syndrome (HUS), the latter representing the main cause of renal impairment in children (Caprioli et al., 2005). STEC infection involves the colonization of the host intestine and the production of Shiga toxin (Stx), a potent cytotoxin with a tropism for the endothelial epithelium, encoded by genes located on lambdoid bacteriophages integrated in the bacterial chromosome in a lysogenic state (O’Brien et al., 1982; Karmali et al., 1983; O’Brien et al., 1984).

In addition to Stx-bacteriophages, STEC strains can increase their pathogenicity by acquiring other virulence features *via* horizontal gene transfer, leading to the emergence of new cross-pathotype strains, as in the case of STEC strains belonging to O80:H2 serotype, first described from a HUS patient with bacteremia in France (Cointe et al., 2018) and characterized by the presence of virulence features typical of Extraintestinal Pathogenic *E. coli* (ExPEC). These hybrid strains have been shown to carry the peculiar mosaic plasmid pR444_A, a pS88-like plasmid typical of ExPEC strain S88, that combines a panel of virulence determinants, including the *hlyF, iro(BCDEN), iss*, and *ompT* genes, with multiple antimicrobial resistance (AMR) genes (Chuba et al., 1989; Dozois et al., 2003; Morales et al., 2004; Peigne et al., 2009; Haiko et al., 2010; Cointe et al., 2018). Among these features, the *hlyF* gene is gaining interest since it is associated with an increased production of outer membrane vesicles (OMVs), as observed in ExPEC strains (Murase et al., 2016).

Outer Membrane Vesicles (OMVs) are spherical structures delimited by a lipidic bilayer membrane that allow bacteria to interact with their environment, enhancing bacterial fitness, delivering virulence factors into host cells and regulating interaction within bacterial communities by transferring genetic material between bacterial cells (Schwechheimer and Kuehn, 2015). Interestingly, it has been observed that OMVs can deliver extracellular RNA molecules, including both structural non-coding RNAs, such as tRNA and rRNA fragments, and RNAs derived from cryptic prophages and intergenic or coding regions, with most of them not characterized yet (Ghosal et al., 2015).

Small RNAs produced by bacteria, described as non-coding RNAs shorter than 200 nucleotides (Ghosal et al., 2015), have been shown to have many regulatory mechanisms, such as the regulation of gene expression by binding to Hfq protein or blocking the ribosome-binding site. Moreover, through OMVs they can be transferred to other microbes or host cells, sometimes altering production and secretion of cytokines (Ahmadi Badi et al., 2020).

Recent studies showed that OMVs produced by STEC strains are internalized by human intestinal epithelial cells and can be translocated across the intestinal epithelial barrier and enter microvascular endothelial cells, which represent the main targets during STEC infection (Bielaszewska et al., 2021). OMVs produced by STEC strains have been demonstrated to act as a secretory system for virulence factors, such as enterohemolysin and Stx (Bielaszewska et al., 2021): bound on the surface or as part of OMV cargo, the virulence factors can reach even long-distance targets, causing cell damage (Ghosal et al., 2015).

In addition to the studies aiming at unravelling the role of OMV as virulence determinants translocators, a few studies have investigated the small RNAs content in bacterial OMVs and particularly those released by *E. coli*, confirming the presence of this genetic material in the vesicles, which could contribute to the communication with the intestinal microbiota and with the host cells (Ghosal et al., 2015; Blenkiron et al., 2016). Nevertheless, information on the content of small RNAs in OMVs produced by STEC is still lacking and could provide interesting insights on the modulation of STEC pathogenesis.

In this perspective, the aim of this study was to identify and characterize the small RNAs carried by the OMVs produced by two *hlyF*-positive STEC strains, which may have role in the interplay of STEC with the intestinal microbiota and host cells during the infection.

## Materials and methods

### Bacterial strains

Two previously described unrelated STEC strains (Gigliucci et al., 2021) harboring virulence genes typical of ExPEC strains, including *hlyF*, were selected for this study among those available in the STEC collection at the European and National Reference Laboratory for *E. coli*, Istituto Superiore di Sanità, belonging to different serotypes and isolated from different sources. In detail, ED1284 strain belonged to O26:H11 serotype and was isolated from a case of HUS in Italy in 2018, while ED1374 strain belonged to O80:H2 serotype and was isolated from a milk sample in Italy in 2019 (Gigliucci et al., 2021) (**Table 1**). The two strains were positive for the presence of *stx2* gene, subtype *stx2a*.

**Table 1:** Bacterial strains and WGS accession numbers of the strains included in genomic comparison for small RNA identification.

Strain	Serotype	Main virulence genes	*stx* subtype	Accession number	Reference
ED1283	O26:H11	*eae, stx2, hlyF*	*stx2a*	ERR4096140	(Gigliucci et al., 2021)
ED1284*	O26:H11	*eae, stx2, hlyF*	*stx2a*	ERR4129084	(Gigliucci et al., 2021)
ED1374*	O80:H2	*eae, stx2, hlyF*	*stx2a*	ERR4129093	(Gigliucci et al., 2021)
ED1382	O80:H2	*eae, stx2, hlyF*	*stx2a*	ERR4129088	(Gigliucci et al., 2021)
ED1386	O80:H2	*eae, stx2, hlyF*	*stx2a*	ERR4096134	(Gigliucci et al., 2021)
MG1655	Orough:H48	–	–	NC_000913	(Blattner et al., 1997)

*used in this study for OMVs preparations.

### Outer membrane vesicles purification

The OMVs produced by ED1284 and ED1374 were purified in separate extractions and in two independent biological replicates as previously described (Bielaszewska et al., 2021), with some modifications. For each strain, single colonies were grown in 500 ml of LB broth overnight at 37°C. The cultures were then subjected to centrifugation at 9.800 x *g* for 10 minutes at 4°C to remove bacterial cells. The supernatants were filtered with 0.22 µm filters and subjected to two sequential steps of ultracentrifugation using Optima XPN-90 Ultracentrifuge and Type 45 Ti rotor (Beckman Coulter, Pasadena, CA, United States) at 100.000 and 200.000 x *g* of two hours each, at 4°C, by collecting the pelleted OMVs in two separate fractions, termed Fraction 1 (FR1) and Fraction 2 (FR2), respectively. Sequential ultracentrifugation was used to eliminate confounding signals from non-vesicular material, that may disturb both qualitative and quantitative downstream investigations, and to ensure that only high-quality OMV preparations (free from significant contamination) would be used for further analyses. The pellets obtained for each fraction of the two strains were resuspended in 900 µl of Tris-HCl 20 mM, pH 8.0. A sterility assay was performed by plating 10 µl of each OMVs preparation on LB agar plates then incubated overnight at 37°C.

### Electron Microscopy and Nanoparticles Tracking Analysis of OMVs preparations

The OMVs preparations were analyzed by Scanning and Transmission Electron Microscopy (SEM and TEM), in order to analyze the surface morphology for a qualitative characterization of the samples and to inspect the lipidic-membrane-enclosed nature of particles, respectively (Behrouzi et al., 2018; Cui et al., 2022; Wu et al., 2025).

For SEM analysis OMVs preparations were left to adhere to polylysine-treated round glass coverslips (10 mm) for 5 h at room temperature. According to Shively and Miller, samples were first fixed with glutaraldehyde 2.5% in sodium cacodylate buffer and then post-fixed with osmium tetroxide 1%. Samples were dehydrated through a graded series of ethanol and hexamethyldisilazane (HMDS) solutions, then subjected to final drying and evaporation for two hours (Shively and Miller, 2009). Dried coverslips were mounted on stubs, gold-sputtered (10 nm) and analyzed by QUANTA INSPECT F (FEI, Hillsboro, Oregon, United States).

For TEM analysis 10 µl of each preparation were deposited on a TEM grid covered with a slight carbon layer, then incubated at room temperature for five minutes. The excess solution was discarded, and the grid left to air dry at room temperature. A 1:1 solution of ammonium molibdate 4%, pH 6.8 and fosfotungstic acid 2% was used as contrasting solution. The prepared samples were observed with a TEM PHILIPS EM208 instrument and a Megaview II SIS camera (Olympus). Nanoparticles Tracking Analysis (NTA) was also performed on the OMVs preparations to measure size and concentration. In detail, each OMVs fraction was analyzed with Nanosight NTA NS300 instrument, software version 3.4 (Malvern Panalytical, Malvern, Worcestershire, United Kingdom) using Blue488 laser with the following parameters: 15 camera level, 5 detection threshold, room temperature, water viscosity, 5 minutes video-acquisitions.

### Genomic analyses

The whole genome sequences of the two tested strains (ED1284 and ED1374) and of three additional STEC strains positive for *hlyF* were recovered from EMBL ENA database (Acc. No. PRJEB38068) (Gigliucci et al., 2021) and analyzed in comparison with those of MG1655 non-pathogenic *E. coli* K12 strain (Blattner et al., 1997) (**Table 1**) and the complete sequence of pR444_A plasmid (Acc. No. NZ_QBDM01000004). The whole genome sequences of the STEC strains were analyzed on ARIES web platform (Istituto Superiore di Sanità, https://www.iss.it/site/aries) (Knijn et al., 2020) by assembling contigs using SPADES v. 3.12.0 with default parameters (Bankevich et al., 2012) and filtering the obtained contigs with Filter Spade’s repeats tool (https://github.com/phac-nml/galaxy_tools) with default parameters. The filtered contigs obtained for the analyzed strains and the complete sequence of pR444_A plasmid were then annotated for the presence of functional elements by using Prokka tool v. 1.14.5 (Seemann, 2014), using default options and enabling searching for non-coding RNAs with Infernal+Rfam module (Nawrocki, 2014; Kalvari et al., 2018). Non-coding RNAs, labelled by the tool as “miscellaneous RNA”, were extracted and filtered for small RNAs by applying a maximum length filter set at 200 bp (Ghosal et al., 2015).

### Detection and quantification of small RNAs in the OMVs by Real Time PCR

The two fractions of OMVs preparations obtained by ultracentrifugation of strains ED1284 and ED1374 were concentrated using Amicon Ultra 100k filters (Merck KGaA, Darmstadt, Germany) by centrifuging at 5.000 x *g* for 30 minutes. In detail, from a starting volume of 800 µl, total 130 µl were recovered per fraction. Prior to the extraction of RNA within OMV, the preparations were stored overnight at 4°C to allow the degradation of any external contaminant RNA, being not protected by the OMV lipid bilayer. The preparations were treated with RNase-Free DNase (Qiagen, Valencia, CA) for one hour at 25°C in order to avoid the presence of any genomic DNA in the samples. Total RNA was extracted from the two independent and concentrated OMVs preparations, using the Total RNA Purification Kit (Norgen Biotek Corp., Torold, Canada), by applying the optional preliminary treatment protocol specific for RNA extraction from exosome preparations. The recovered RNA was quantified with Qubit RNA HS Assay kit on a Qubit 2.0 Fluorometer (Thermo Fisher Scientific, Waltham, MA, USA) and reverse transcribed using QuantiTect Reverse Transcription kit (Qiagen, Valencia, CA), by performing a preliminary Genomic DNA Wipeout step to eliminate genomic DNA before reverse transcription, following manufacturer instructions. The obtained cDNA was quantified with Qubit dsDNA HS Assay kit on a Qubit 2.0 Fluorometer (Thermo Fisher Scientific, Waltham, MA, USA) and tested for the presence of the putative small RNAs identified in the respective genomic sequences by Real Time PCR, by using the SensiMix SYBR No-ROX kit (Bioline, Meridian Life Science, Memphis, TN, USA) and specifically developed primer pairs (**Table 2**). For each reaction, melting curve analysis was performed to check that only specific amplification was obtained. Each primer was used at a final concentration of 20 µM. For putative small RNAs identified in multiple slightly different copies in the genomic sequences, primer pairs specific for each target were deployed by using Primer-BLAST tool applying the “Primers common for a group of sequences” option, to develop primer pairs able to detect all the different copies of each small RNA identified in the genomic sequences of strains ED1284 and ED1374. The amplification products were confirmed by Sanger sequencing (Eurofins Genomics, Eurofins, Ebersberg Germany).

**Table 2:** Primers pairs developed in this study targeting small RNAs identified in the genome of *hlyF*-positive STEC strains.

Small RNA	Primers sequences (5’-3’)	Annealing and Extension Temperature	Amplicon length (bp)
ALIL	F: TTCGACCGCCTCTGGAAAAA R: GGGCAATATGCAGTTCGCTG	56°C	93
AS-pc01	F: ACACAGGTTAGTGAAGCCGT R: GGTCAGGGACCTGCTTAATCC	56°C	71
AS-pc02	F: TCAGCCTTCTGAATCCCTGG R: CCCGAAAAAGGGCTGAACAC	58°C	93
CopA	F: GGCTATACGGTTTAAGTGGGC R: AGCAAAAACCCCGATAATCTTCT	56°C	76
EFASI	F: CAGGCAGATATATCGGGGGC R: TTCAGGTTTAGCGGGGTGTC	58°C	89
GroupII-D1D4-2	F: CAGGCCCACCGGGATCT R: CCATGGCTCTTTACCACAGC	58°C	70
GroupII-D1D4-3	F: AAGCCGGATGCAAAATGCTG R: GCGCGACTTTCCTGCTTATG	56°C	142
GroupII-D1D4-7	F: GGTAACGGTGGGGTGTGAAG R: AACCTCACGGTTTCATGCGA	58°C	70
Intron_gpII	F: GAAAGGTGCACGTCCGGTTC R: TAGAGTAAGAGGCAGGGCGT	58°C	70
PK-repZ	F: TCTCATCGAACTTGGCGGAA R: CGTGCGTTAATACACTGCGA	56°C	112
STAXI	F: TTTGCGACCAGCCAATGTTC R: AAGGCATCCTCTTTCAGGCT	56°C	131
STnc_100	F: CATGGTGAATCCCCCTGTGC R: GCCTCCCGGTGAATTCAGTA	58°C	93

To measure the concentration of the small RNAs, absolute quantitative Real Time PCR experiments were performed in three technical replicates and mean and standard deviation values were recorded. DNA fragments amplified through conventional PCR on genomic DNA preparations were used to build standard curves. In detail, total DNA to use as template was extracted using GRS DNA extraction kit (GRiSP, Porto, Portugal) following manufacturer’s instructions and the same primer pairs and conditions used for Real Time PCR assays were employed (**Table 2**) with BIOTAQ™ DNA polymerase (Bioline, Meridian Bioscience, Cincinnati, Ohio) amplification kit. The obtained products were subjected to DNA extraction from agarose gel electrophoresis (QIAquick Gel Extraction kit, Qiagen, Valencia, CA), quantified using Qubit dsDNA HS Assay kit on a Qubit 2.0 Fluorometer (Thermo Fisher Scientific, Waltham, MA, USA) and diluted with serial dilutions 1:10 to serve as templates for standard curves preparation. The concentration of each target small RNA in each preparation was estimated by calculating the number of copies per ng of analyzed cDNA. Statistical significance was calculated for the differences in STnc_100 target concentration between cDNA obtained from FR2 of the two strains, by applying unpaired T-test.

### Structural and functional analysis of the small RNAs detected in the OMVs

The RNAfold server (http://rna.tbi.univie.ac.at/cgi-bin/RNAWebSuite/RNAfold.cgi) was used to predict the secondary structure of the small RNAs identified in the OMVs. The RFAM platform (https://rfam.org/) was searched to retrieve available information about the function and distribution of the identified small RNAs. Homologous sequences to those of the detected small RNAs were compared by using NCBI BLAST+ blastn tool (v. 2.14.1) on ARIES webserver against the latest available version of the NCBI nucleotidic sequences database (January 25^th^, 2024) with default parameters. The following filters were applied to the results: at least 80% of sequence identity, at least 50% of sequence length aligned (Knijn et al., 2020). Finally, the localization of the sequence of STnc_100 in the genome of the two strains was analyzed by inspecting the annotated contigs obtained through Prokka as described above and the platform Freiburg RNA tools-Inta RNA (https://rna.informatik.uni-freiburg.de/) to look for complementarity regions among the sequence of STnc_100 small RNA and that of *stxAB* operon.

We have submitted all relevant data of our experiments to the EV-TRACK knowledgebase (EV-TRACK ID: EV250058) (Van Deun et al., 2017).

## Results

### Characterization of the OMVs produced by the two *hlyF*-positive STEC strains

**Figure 1:**
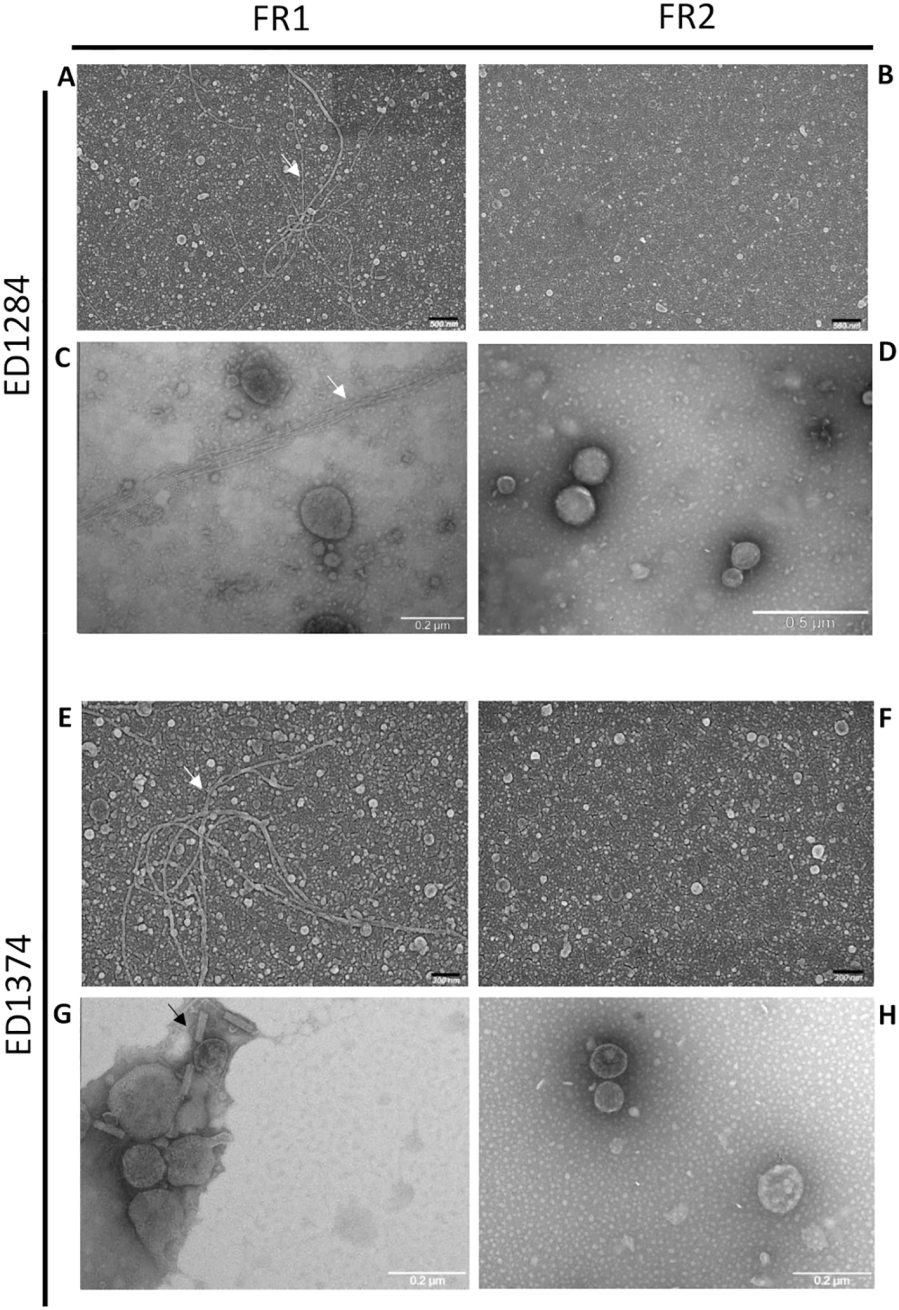
Representative images of SEM **(A, B, E, F)** and TEM **(C, D, G, H)** acquisitions of FR1 and FR2 obtained from both ED1284 and ED1374 strains. The analyses were conducted once per preparation. Debris, bacterial pili (white arrows) and phages (black arrows) were observed in FR1 of both strains.

SEM analysis showed preparations consisting of vesicles well dispersed with different sizes (**Figures 1A, B, E, F**), while TEM characterization confirmed the lipidic-membrane-enclosed nature of particles of two fractions obtained for both the strains assayed ED1284 and ED1374 (**Figures 1C, D, G, H**). Electron microscopy observation of the two fractions revealed that FR1, in addition to outer membrane vesicles (OMVs), contained a high abundance of cellular debris and contaminants, including bacterial pili, flagella, and phages in both examined strains (**Figures 1A-C, E-G**; **Supplementary Figure 1**). In contrast, FR2 of ED1284 and ED1374 was enriched in vesicles and exhibited reduced levels of debris and contaminants.

**Figure 2:**
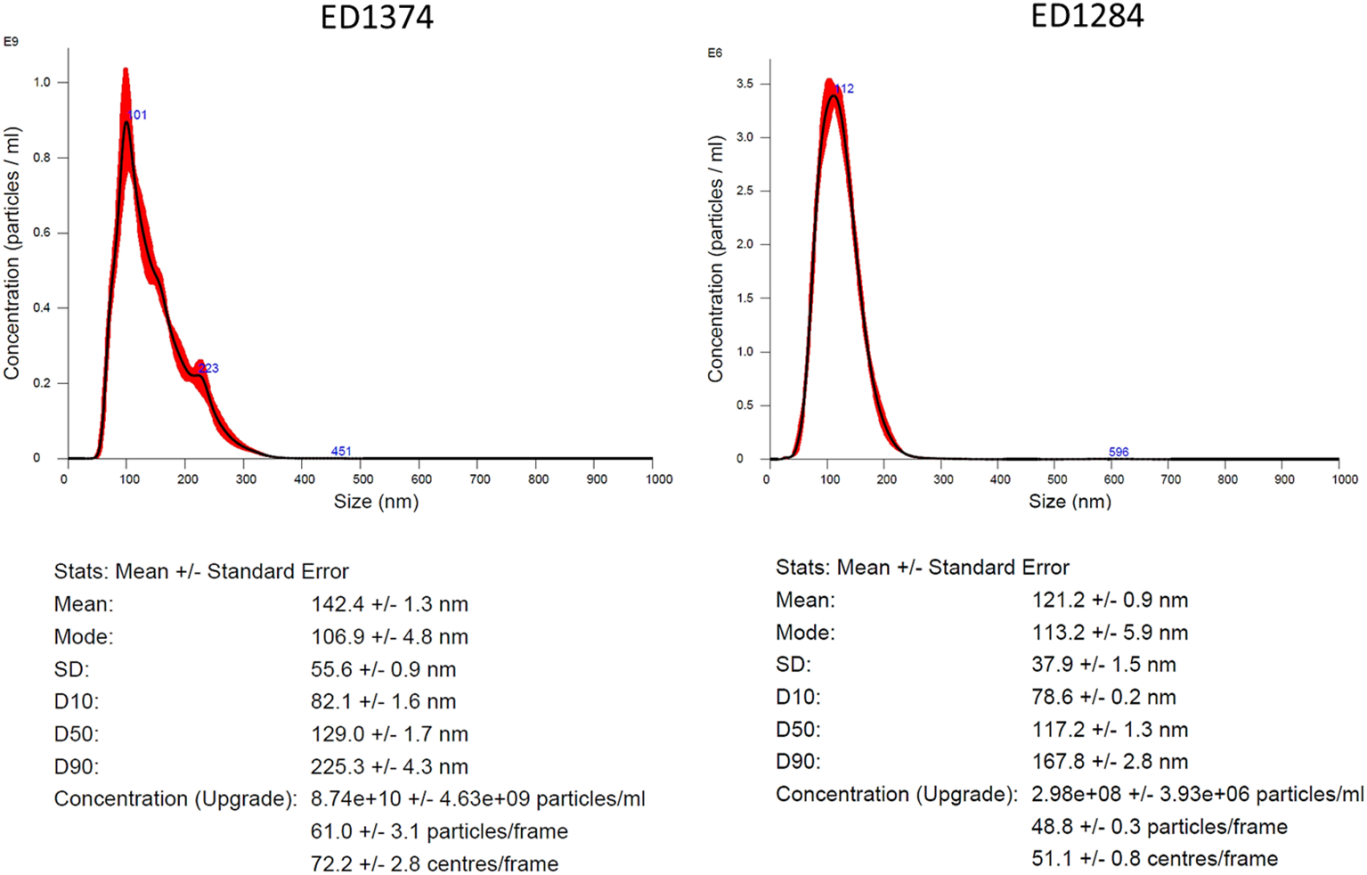
Characterization of isolated OMVs by NTA. Representative graph depicting the hydrodynamic size distribution profiles of isolated OMVs from FR2 of ED1374 (on the left) and of ED1284 strain (on the right). The measurements were performed once per preparation.

Size and concentration of OMVs isolation in FR2 was measured by NTA (**Figure 2**), while in FR1 noise was too high for a reliable measure (data not shown). OMVs isolation from both strains resulted in quite homogeneous FR2 preparations. ED1284 showed a single peak with very similar mode and mean, 113.2 ± 5.9 nm and 121.2 ± 0.9 nm respectively, and a concentration of about 3 x 10^8^ ± 4 x 10^6^ particles/ml. The strain ED1374 showed a two-orders higher concentration, about 9 x 10^10^ ± 4.6 x 10^9^ particles/ml, and a slightly wider distribution resulting in a mode of 106.9 ± 4.8 nm and a mean of about 142.4 ± 1.3 nm.

Taken together, SEM, TEM, and NTA data allowed us to validate the efficacy of sequential centrifugation in discriminating between the two fractions of OMVs produced by both strains and to select the most suitable one for downstream studies. In both strains, FR1 showed an abundance of unwanted non-OMV materials, which could interfere with downstream investigation (Welsh et al., 2024). On the other hand, FR2 isolated from both strains showed an acceptable depletion of such contaminants, resulting in much homogenous and measurable OMV-enriched preparations.

Based on these results, the use of sequential ultracentrifugation steps represented an optimization of the isolation method, recommended for separating distinct fractions of OMVs to overcome the limitations of ultracentrifugation (Nawrocki, 2014). As FR1 was shown to contain unwanted non-OMV material, we selected only FR2 from both strains for the study of small RNAs content within OMVs.

### Identification of small RNAs sequences in the genomes of *hlyF*-positive STEC strains

Putative small RNAs were predicted by analyzing the whole genome sequences of five *hlyF*-positive strains, including the two *hlyF*-positive STEC strains under study, and screening out molecules longer than 200 bp in length, in order to fit small RNAs definition (Ghosal et al., 2015), excluding also those found in the genome of MG1655 *E. coli* K12 strain, with the aim of focusing on STEC-specific small RNAs. A total of 27 putative small RNAs were identified in the genomic sequences of at least one of the five *hlyF*-positive strains (**Table 3**). Ten of these were also identified in the sequence of pR444_A plasmid, harboring *hlyF* gene in RDEx444 reference strain (Cointe et al., 2018), suggesting that these may be harbored by similar plasmids also in the analyzed genomes. The identification of 27 putative small RNAs in the genome of *hlyF*-positive STEC strains, supported the idea that possibly regulatory elements might be carried within OMVs.

**Table 3:** Putative small RNAs identified on *hlyF*-positive STEC genomes and absent in the genome of *E. coli* K12 MG1655 strain.

Small RNA	ED1283	ED1284	ED1374	ED1382	ED1386
AAC_AAD_leader*	+	+	+	+	+
AgvB	-	-	+	-	+
ALIL*	+	+	+	+	+
AS-pc01*	+	+	-	+	+
AS-pc02*	+	+	+	+	+
AS-traG*	+	+	+	+	+
AsxR	+	+	+	+	+
C4	+	+	+	+	+
CopA*	+	+	+	+	+
EFASI	+	+	-	+	-
Esr41	+	+	+	+	+
FinP*	+	+	+	+	+
group-II-D1D4-1	-	-	+	+	+
group-II-D1D4-2	-	+	-	+	+
group-II-D1D4-3	-	-	+	+	+
group-II-D1D4-7	-	-	+	+	+
int-alpA	-	-	-	+	+
Intron_gpII*	-	-	+	+	+
IS009	+	+	+	+	+
Lambda_thermo	+	+	-	-	-
PK-repZ	+	+	+	+	-
RNAI	-	-	-	-	+
sRNA-Xcc1	+	+	-	-	-
STAXI	+	+	-	+	-
STnc_100	+	+	+	+	+
STnc_490*	+	-	+	+	+
traJ_5*	+	+	+	+	+

*also identified in the sequence of pR444_A plasmid.

### Identification and quantification of small RNAs in the OMVs

Primer pairs specific to the putative small RNAs identified through genomic analyses were deployed to test the presence and concentration of these targets in OMVs preparations obtained from the ED1374 and ED1284 test strains. Two out of the 27 putative small RNAs (int-alpA and RNAI) were not detected in the genomic sequences of the two test strains (**Table 3**) and thus were not further investigated. Additionally, due to the short size of the small RNAs, it was not possible to design primer pairs targeting all the remaining putative small RNAs. The primer pairs finally developed targeted 12/25 putative small RNAs (**Table 2**). Specific real Time PCR assays allowed to detect the presence of nine of the 12 tested putative small RNAs in the cDNA preparations obtained from OMVs of at least one of the two strains (**Table 4**). Three of these, namely ALIL, As-pc01 and As-pc02, were among those identified in the sequence of pR444_A plasmid. Interestingly, the STnc_100 small RNA was the only one identified in the OMVs preparations collected by both the strains.

**Table 4:** Small RNAs identified inside the OMVs produced by *hlyF*-positive STEC strains through Real Time PCR.

Small RNA	ED1284	ED1374
ALIL*	+	-
As-pc01*	+	NA
As-pc02*	-	+
CopA*	-	-
EFASI	+	NA
GroupII-D1D4-2	+	NA
GroupII-D1D4-3	NA	-
GroupII-D1D4-7	NA	+
Intron_gpII*	NA	-
PK-repZ	+	-
STAXI	+	NA
STnc_100	+	+

*also identified in the sequence of pR444_A plasmid. NA: not applicable; small RNA absent in the genome of the test strain from WGS analysis.

**Figure 3:**
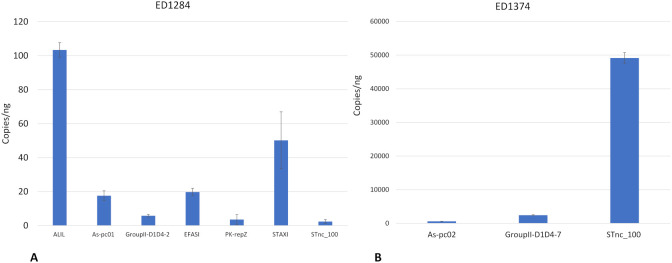
Concentration in copies/ng of small RNAs in FR2 of OMVs preparations obtained from strain ED1284 **(A)** and from strain ED1374 **(B)**. The experiments were conducted in three technical replicates. Mean values are shown, with error bars representing the standard deviation of the data.

Absolute quantitative Real Time PCR assays were applied in triplicate on OMVs preparations obtained from both strains, in order to estimate the concentration of the small RNAs in the OMVs. In detail, the reactions were performed on cDNA preparations obtained from FR2. The results obtained for strain ED1284 and ED1374 are reported in **Figure 3**, showing mean and standard deviation values obtained.)STnc_100 target, the only small RNA identified in both fractions of both strains, showed a much higher abundance in FR2 of ED1374 strain (49.145,9 ± 1.585,2 copies/ng) compared to FR2 of ED1284 strain (2,36 ± 1,15 copies/ng) and such difference was shown to be statistically significant (p < 0,0001).

To investigate a possible differential compartmentalization of the targets in the two fractions, and an eventual loss of targets in FR1, absolute quantitative Real Time PCR assays were also performed on FR1 preparations obtained by both strains. In both cases, the small RNA targets previously identified in FR2 were also present in FR1 (**Supplementary Figure 2**).

These results suggested that the selection of the only FR2 did not cause a loss of interesting genetic material (i.e. small RNAs), since the same small RNAs were identified in both FR1 and FR2. Of the nine small RNAs identified and quantified in the OMVs produced by the two test strains, only STnc_100 was present in both, with a significative higher concentration in ED1374-FR2 with respect to ED1284-FR2.

### Characterization of the small RNAs harbored by OMVs: function, secondary structure and sequence homologies

The predicted secondary structures of the identified small RNAs and the corresponding free energy values ​​are shown in **Supplementary Figure 3**.

The information about the distribution and mechanisms of action of the identified small RNAs obtained from the RFAM platform are reported in **Table 5**. For many of the small RNAs identified, a regulatory function in the processes of DNA replication, integration and transposition and in the stress response has been hypothesized.

**Table 5:** Functions of small RNA identified in OMVs isolated from ED1374 and/or from ED1284 strains derived from RFAM.

Small RNA	Information about function and distribution of small RNAs from RFAM
GroupII-D1D4-2	Group II introns are a large class of self-catalytic ribozymes and mobile genetic elements found within the genes of all three domains of life.
GroupII-D1D4-7	
EFASI	The Enterobactericaea, Frequently Around STAXI and Integrases (EFASI RNA motif) is a conserved RNA structure that was discovered by bioinformatics. EFASI motifs are found in some organisms in the lineage Enterobacteriaceae. EFASI RNAs likely function in *trans* as small RNAs. Many EFASI RNAs are found several hundred base pairs downstream of STAXI RNA motif examples and upstream of integrase-encoding genes. Although this association likely relates to the function of EFASI RNAs, this function is not yet known.
PK-repZ	The regulatory region of the *repZ* gene, which encodes the replication initiator of plasmid ColIb-P9, contains a pseudoknot. This acts as a molecular switch controlling translation of *repZ* and *repY*.
STnc_100	Small RNAs typical of Gammaproteobacteria.
STAXI	The Ssbp, Topoisomerase, Antirestriction, XerDC Integrase RNA motif (STAXI RNA motif) is a conserved RNA-like structure identified using bioinformatics. STAXI RNAs are located near to genes encoding proteins that interact with DNA (Ssbp, topoisomerase, XerDC integrase) or are associated with such proteins (antirestriction proteins, which inhibit restriction enzymes). This observation raised the possibility that instances of the STAXI motif function as single-stranded DNA molecules, perhaps during DNA replication or DNA repair.
As-pc01 *	Antisense RNAs to genes involved in plasmids replication, conjugal transfer and stabilization.
As-pc02 *	
ALIL *	ALIL pseudoknot is a RNA element that induces frameshifting in bacteria, stimulating the expression of transposase. It’s conserved in several bacterial species.

*= also identified on pR444_A plasmid.

The comparison of the sequences of the identified small RNAs with the non-redundant database highlighted the presence of homologous regions in the genomes of many bacterial species belonging to the *Enterobacteriaceae* family, including the *E. coli* species itself and species belonging to the genera *Klebsiella, Proteus, Salmonella* and *Shigella* (**Table 6**). Regions homologous to the targets have also been found in bacterial species belonging to different families, as in the case of *Pseudomonas aeruginosa*, belonging to the *Pseudomonadaceae*, and several species belonging to *Burkholderiaceae* family, or in bacterial species that are the causative agents of plant’s infections, such as *Xanthomonas* spp., and *Agrobacterium vitis*. In addition to bacteria, it was possible to identify homologous regions to some of the targets also in bacteriophage sequences, including Stx-converting phages, which were further investigated.

**Table 6:** List of bacterial species and microorganisms in which regions homologous to the small RNA sequences identified in the OMVs of ED1284 and/or ED1374 strains were found.

Target name	Species in which the target is present
GroupII-D1D4-2	*Escherichia* spp. *Shigella boydii* *Shigella flexneri* *Shigella sonnei*
GroupII-D1D4-7	*Achromobacter xylosoxidans* *Agrobacterium vitis* *Alcaligenes* spp. *Alkaliflexus* spp. *Anaerovoracaceae bacterium* *Bacteroidales bacterium* *Bordetella petrii* *Bradyrhizobium* spp. *Burkholderia anthina* *Burkholderia latens* *Burkholderia* spp. *Cabelleronia* spp. *Enterobacter kobei* *Escherichia fergusonii* *Klebsiella michiganensis* *Klebsiella pneumoniae* *Marimonas* spp. *Mucilaginibacter gotjawali* *Neorhizobium petrolearium* *Paraburkholderia caribensis* *Paraburkholderia hospital* *Paraburkholderia kirstenboschensis* *Paraburkholderia phymatum* *Paraburkholderia terrae* *Prolixibacteraceae bacterium* *Proteus mirabilis* *Pseudomonas aeruginosa* *Pseudomonas amygdali* *Rhizobium tropici* *Salmonella enterica* *Shigella boydii* *Shigella dysenteriae* *Sphingomonas sanxanigenens* *Thiothrix fructosivorans* *Xanthomonas* spp.
STAXI	*Escherichia* spp.
EFASI	*Escherichia* spp.
PK-repZ	*Escherichia fergusonii* *Escherichia marmotae* *Escherichia* spp. *Klebsiella pneumoniae* *Salmonella enterica* *Shigella flexneri* *Shigella sonnei* *Shigella* spp.
STnc_100	*Enterobacteria phage* *Escherichia albertii* *Escherichia phage* *Escherichia* spp. *Shigella boydii* *Shigella dysenteriae* *Shigella flexneri* *Shigella sonnei* *Shigella sonnei* *Stx-converting phage*
As-pc01 *	*Escherichia albertii* *Escherichia fergusonii* *Klebsiella pneumoniae* *Pseudomonas aeruginosa* *Salmonella enterica* *Shigella dysenteriae* *Shigella flexneri*
As-pc02 *	*Escherichia albertii* *Escherichia fergusonii* *Escherichia* spp. *Klebsiella oxytoca* *Klebsiella pneumoniae* *Pseudomonas aeruginosa* *Salmonella enterica* *Shigella dysenteriae* *Shigella flexneri* *Shigella sonnei*
ALIL *	*Escherichia albertii* *Escherichia fergusonii* *Escherichia phage* *Escherichia* spp. *Klebsiella pneumoniae* *Proteus mirabilis* *Salmonella enterica* *Shigella boydii* *Shigella dysenteriae* *Shigella flexneri* *Shigella sonnei* *Stx-converting phage*

*= also identified on the pR444_A plasmid.

Our results showed that the identified small RNAs have a predicted stable secondary structure, putatively performing crucial regulatory functions for metabolism and fitness, are conserved in a wide range of bacteria, and in some cases, in phages, suggesting evolutionary relevant and potentially shared roles.

### Localization of the small RNA STnc_100 on Stx-bacteriophage

**Figure 4:**
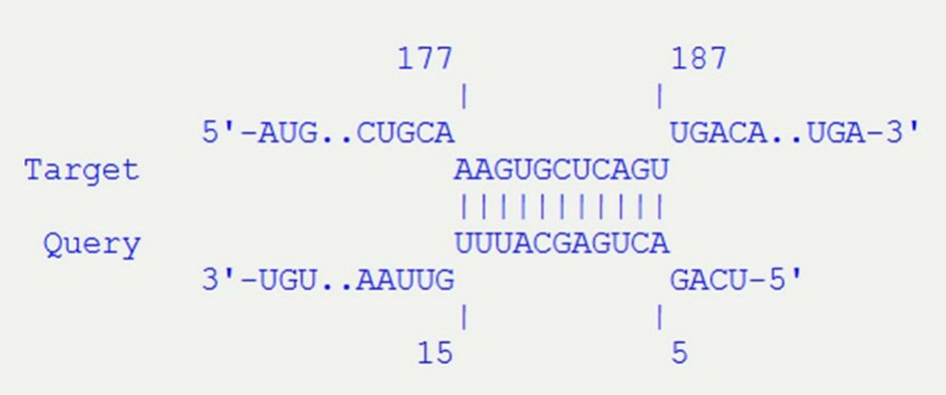
Details of the interaction of STnc_100 (Query) with *stxB* gene (Target), predicted with IntaRNA tool. The sequences of STnc_100 and *stxB* are 100% identical in the two strains analyzed (ED1284 and ED1374).

In particular, by inspecting the contigs assembled for the genomes of ED1284 and ED1374 strains, we found that one copy of the STnc_100 target, identified within the OMVs of both *hlyF*-positive STEC strains analyzed, is encoded on Stx2-encoding phages 75 nucleotides downstream of the *stx2* operon in both strains (**Figure 4**). Interestingly, an exact complementarity of 11 nucleotides between STnc_100 and *stxB* gene was found (positions 5–15 in the sequence of STnc_100 small RNA corresponding to positions 177–187 in *stxB* gene), suggesting a regulatory function in Stx production. Such complementarity had an estimated free energy of -10.69 kcal/mol.

This evidence showed that STnc_100, the only small RNA identified in the OMVs produced by both strains, is harbored by Stx2-phages, and suggests that it may exert a role in the modulation of the Stx release through the affinity with *stxB* gene.

## Discussion

The ability to generate and deliver OMVs is evolutionarily conserved among bacteria, playing diverse biological and pathophysiological functions (Jan, 2017). As regards OMVs produced by STEC strains, they have been shown to be internalized by human intestinal epithelial cells and endothelial cells of the microvasculature, which represent respectively the transfer pathway and the target cells of Shiga toxins during STEC infection (Park et al., 2010; Rueter and Bielaszewska, 2020; Bielaszewska et al., 2021). Interestingly, a recent study revealed the presence in the genome of some STEC strains of the *hlyF* gene, a factor carried by the pR444_A plasmid, described in ExPEC strains and associated with increased OMVs production (Murase et al., 2016; Gigliucci et al., 2021). It has been demonstrated that STEC-OMVs can contribute to the pathogenesis of STEC carrying a cocktail of virulence factors, such as Shiga toxin 2a (Stx2a), cytolethal distension toxin type V (CdtV) and enterohemolysin, but also flagellin, lipopolysaccharide and nucleic acid molecules (Murase et al., 2016; Rueter and Bielaszewska, 2020). Among these, the small RNAs harbored by OMVs have attracted particular attention since they could represent mediators in intercellular communication between different bacterial species and between bacteria and host cells. Within OMVs, the RNAs are protected from the action of RNases present in the extracellular environment, suggesting that this OMV-mediated transport would allow them to reach the target cells in an intact and active form and, potentially, to modulate the infection process (Ahmadi Badi et al., 2020). However, information about the RNA content of OMV produced by STEC strains is still scanty.

For this reason, in this work we characterized the OMVs and investigated the small RNA content of OMVs produced by two *hlyF*-positive ExPEC/STEC hybrid strains, ED1284 and ED1374, both producers of Stx2a, but belonging to two different serotypes and isolated from human and animal sources, respectively (Gigliucci et al., 2021). These cross-pathotype strains ExPEC/STEC were selected for this study since the *hlyF* gene has been associated with an increased production of outer membrane vesicles (OMVs), observed in ExPEC strains (Murase et al., 2016).

As for the purification of OMVs, ultracentrifugation may co-isolate and/or aggregate unwanted non-OMV materials including bacterial pili, flagellae, phage, protein, lipoprotein, and nucleoprotein complexes, that may affect their measurement by downstream assays (i.e. electron microscopy and optical analysis methods) (Welsh et al., 2024). Our findings demonstrate that the use of two different subsequent ultracentrifugation speeds, instead of a single ultracentrifugation at 235,000 x *g* as previously described in literature (Bielaszewska et al., 2021), allowed us to select the FR2 as the fraction most enriched in vesicles and poorest in different kind of contaminants (i.e. protein agglomerates, salts, fimbria, phages, etc…) with respect to FR1 as demonstrated by SEM and TEM observations (**Figure 1**). Based on these results, FR2 was selected as the most suitable to perform our further investigations.

To deeply characterize and assess the particle size and concentration of the obtained vesicles, we performed NTA on FR2 of OMVs isolated from ED1284 and ED1374 strains. Our results showed that the samples had uniform peaks of particle size distribution (**Figure 2**). Interestingly, the OMVs resulted in about 300 times more concentrated preparations for ED1374 strain. This difference in concentration is suggestive of a higher production of OMVs for the latter strain. However, even if it has been demonstrated that an augmented production of vesicles may lead to an augmented release of toxins in Extraintestinal pathogenic *E. coli* (Murase et al., 2016; Chagneau et al., 2023), we have no evidence to support a higher virulence for ED1374 strain. As a matter of fact, the virulence profile of the two strains (harboring *stx2a*, *eae* and *hlyF* genes) has been associated to severe disease regardless of OMVs production. Moreover, ED1284 strain was isolated from an HUS case, while ED1374 strain was isolated from a food sample. More investigation would be needed for investigating the role of higher production of OMVs in the virulence of STEC strains.

The investigation of sequences of small RNAs on the complete genomic sequences of five *hlyF*-positive STEC strains, including ED1284 and ED1374, allowed the identification of 27 putative small RNAs, present in at least one of the five STEC strains analyzed and absent in the genome of the non-pathogenic *E. coli* K12 strain MG1655. This result suggests that they may be conserved in STEC strains and have a role in the pathogenesis mechanism. Interestingly, 10 of these small RNAs were also present in the reference pR444_A plasmid sequence, suggesting they may be conveyed by the described pR444_A-like plasmids possessed by the test strains (Gigliucci et al., 2021) (**Table 3**). It is known that the pR444_A-like plasmids host several virulence genes, including *hlyF, iro*(BCDEN), *iss* and *ompT*, as well as multiple antimicrobial resistance (AMR) determinants (Murase et al., 2016; Gigliucci et al., 2021). Thus, the co-localization of sequences of these small RNAs on the same plasmid could indicate that they may be involved in the regulation of such genes, but further studies are necessary to confirm this hypothesis.

For 12 of the 27 small RNAs identified (**Table 3**) it was possible to develop targeted Real Time PCR assays, which allowed to identify nine of these small RNAs within the OMVs produced by at least one strain (**Table 4**), three of which putatively associated with pR444_A-like plasmids. This result confirmed the hypothesis of a role of OMVs produced by STEC strains in the delivery of small RNAs, which had never been explored in depth before. On the other hand, the remaining small RNAs targets, whose presence was not confirmed within the OMVs, may be produced by the bacteria and play a regulatory role inside the bacterial cells or be secreted with an OMV-independent secretion mechanism. The small RNAs identified in FR2 fractions could be detected also in FR1 fraction of the same strains, even if in some cases at different concentrations, especially for ED1284. Such differences may be attributed to preferential selection of specific small RNAs during OMV biogenesis, a process that can vary among bacterial strains. In contrast, these differences were not observed in ED1374 strain, suggesting that, under the same growing conditions, RNA composition in OMVs can be induced by strain-specific factors, such as distinct regulatory systems or variation in the composition of the RNA degradosome (Malabirade et al., 2018). Nevertheless, we showed that FR1 fractions were not clean enough from non-OMVs material to allow a proper quantitative comparison among the two fractions.

Interestingly, of the nine small RNAs identified in OMVs, only the STnc_100 target was present in the vesicle preparations obtained from both strains examined, but with different relative abundances. This suggests that the expression of these small RNA or their secretion through OMVs could be regulated differently in different strains, that STnc_100 could play a role in the pathogenesis and that this role could be mediated by OMVs delivery. Nevertheless, our results are based on the detection and characterization of the small RNAs and not on functional validation. Thus, future functional analyses to confirm small RNA regulatory roles are needed to consolidate this preliminary evidence.

The functional prediction obtained by querying the RFAM platform highlighted mainly a predicted regulatory function in the processes of DNA replication, integration and transposition and in the response to stress for the small RNAs identified within the vesicles (**Table 5**). Some of these small RNAs appear to be co-localized at the genomic level, as in the case of the small-RNA EFASI and STAXI. In fact, EFASI (Enterobacteriacae, Frequently Around STAXI and Integrases) is generally encoded by regions downstream those coding the small RNA STAXI (Ssbp, Topoisomerase, Integrase, etc). These two small RNAs may have been conserved together evolutionarily for functional purposes. On the other hand, both were identified among those with highest concentrations in OMVs produced by ED1284 strain (**Figure 3A**). The small RNA STnc_100 had already been described as a small RNA typical of Gammaproteobacteria (Boutet et al., 2022). This study allowed to identify its presence in the genome of STEC strains, in addition to its demonstrated presence in other pathogenic bacterial species (Boutet et al., 2022), and to clarify its secretion via OMVs. Particularly, a recent study showed the presence of multiple copies of STnc_100 in *Salmonella* Tiphymurium LT2 genome, with a binding pattern for RpoD and RpoN sigma factors, highlighting its potential involvement in transcriptional regulation (Lee et al., 2021). Although its actual role remains unclear, a similar function cannot be excluded for STEC strains. The small RNAs termed GroupII-D1D4–2 and GroupII-D1D4–7 are known to be widespread in Archaea, Prokaryotes and Eukaryotes and through OMVs they might play a role in inter-kingdom communication during the infection process. The movement of these small molecules via OMV suggests a possible role in the modulation of physiological processes and horizontal gene transfer of intestinal microbiota during the host infection phase, which could influence the permanence of the pathogen in the intestine and/or the spread of mobile genetic elements carrying virulence or antibiotic resistance genes.

The analysis of the predicted secondary structures (**Supplementary Figure 3**) of the identified small RNAs described structures characterized by numerous hairpins, associated with minimum free energy values ​​between -77.10 and -34.30 kcal/mol, identified as the most probable conformations on a thermodynamic level for these molecules at 37°C (Zuker and Stiegler, 1981). Such structures could allow these small RNAs to avoid degradation during transport in OMVs.

Finally, the search in sequence databases performed through the BLASTn tool allowed us to identify the presence of sequences homologous to those of these small RNAs in the genomes of other bacterial species, which could either encode homologous small RNAs or constitute targets of regulation by the same small RNAs through homologous pairing (**Table 5**). In particular, the presence of regions homologous to the targets of interest was observed in many bacterial species belonging to the *Enterobacteriaceae* family, which also includes the same *E. coli* species, including species belonging to the genera *Klebsiella, Proteus, Salmonella* and *Shigella*. Strains belonging to these species may share the same ecological niche with STEC strains and could therefore be involved in horizontal gene transfer mechanisms mediated by small RNAs transported by OMVs or be influenced in the regulation of gene expression by the same small RNAs. Regions homologous to the targets have also been found in bacterial species that also have hosts other than humans, such as *Xanthomonas* spp. and *Agrobacterium vitis*, which cause various infections in plants. *Ad hoc* studies would be necessary to understand whether these species are also capable of producing and releasing homologous small RNAs or whether they could be targets of the action of small RNAs produced by STEC and released by OMVs in an ecological niche other than the intestine of the host, such as in plants.

Interestingly, it was possible to identify homologous sequences to those of the small RNAs termed ALIL and STnc_100 also in the genome of Stx-converting phages. Indeed, for both ED1284 and ED1374 strains, a copy of STnc_100 was found in the same contig harboring *stx* genes downstream of the sequence coding the Stx2B subunit of Shiga toxin. The STnc_100 target is of particular interest because it was identified within the OMVs of both *hlyF*-positive STEC strains analyzed. Moreover, the identification of an exact match of its sequence with a portion of *stxB* gene suggests a possible role for this small RNA in regulating the production of the toxin, which could be mediated by OMVs delivery. However, the proposed role of STnc_100 in regulating *stx2* gene remains hypothetical. Further analyses, such as *in vitro* reporter assays or RNA-mRNA interaction studies, would be needed to verify whether STnc_100 influences *stx2* expression and contributes to the modulation of STEC infection.

As for the other small RNAs identified in OMVs, currently, there is no evidence reporting their presence in *E. coli* vesicles, including those from STEC strains. On the other hand, in *Salmonella enterica*, several known small RNAs, such as ISrM, CsrC and SsrS have been confirmed within OMVs and are implicated in controlling pathogenicity island-encoded virulence factors, with documented impact on mRNA stability, motility, stress response and host cell invasion (Malabirade et al., 2018). This evidence, together with the existence of Stx-modulating small RNAs (i.e. StxS, DicF) in *E. coli*, although not found within OMVs (Sy and Tree, 2021), underscores the biological plausibility of STnc_100 playing a similarly regulatory role, through vesicle-mediated transfer, which would be worth of further investigation.

This work describes for the first time small RNAs within OMVs released by STEC strains and the identification of homologous sequences in the genomes of bacterial strains also belonging to species other than *E. coli*. The results obtained suggest a possible regulatory role in the mechanisms of Shiga toxin production and horizontal gene transfer between bacterial strains or of inter-kingdom communication, which could influence the production and spread of virulence genes and the colonization of the host. Incorporating additional biological replicates would enable a more comprehensive evaluation of the variability in small RNA content within OMV preparations. Nonetheless, the high degree of consistency observed among the current replicates provides robust support for the presence of the identified targets.

Further in-depth studies on the functions of these small RNAs, on their distribution in other STEC strains and in other bacterial species and on the mechanisms of action, will be useful to investigate their role in the pathogenesis of infections, but also to better understand the ecology of these pathogens in natural reservoirs and during infection.

## Data Availability

The datasets presented in this study can be found in EVtrack online repository under the ID EV250058.

## Supplementary material

Supplementary Figure 1 includes the representative images of TEM **(A-D)** micrograph of FR1 obtained from both ED1284 and ED1374 strains, showing the presence of bacterial pili (white arrows) and phages (black arrows).

Supplementary Figure 2 includes the comparison of small RNAs concentrations in FR1 and FR2 of OMVs preparations obtained from ED1284 and ED1374 strains.

Supplementary Figure 3 shows the representation of secondary structures obtained by RNAfold and their minimum free energy values of the smallRNAs identified within the OMVs of both ED1284 and ED1374 strains.
