# Supplementary material for: Characterization of the small RNAs carried by outer membrane vesicles produced by hlyF-positive Shiga toxin-producing Escherichia coli
Source: Front Cell Infect Microbiol. 2025 Aug 8;15:1621341. doi: 10.3389/fcimb.2025.1621341 (PMC12370729; doi:10.3389/fcimb.2025.1621341)
Supplement: Supplementary Figure 1 — includes the representative images of TEM (A-D) micrograph of FR1 obtained from both ED1284 and ED1374 strains, showing the presence of bacterial pili (white arrows) and phages (black arrows). [file DataSheet1.docx]

**Supplementary Materials**

**
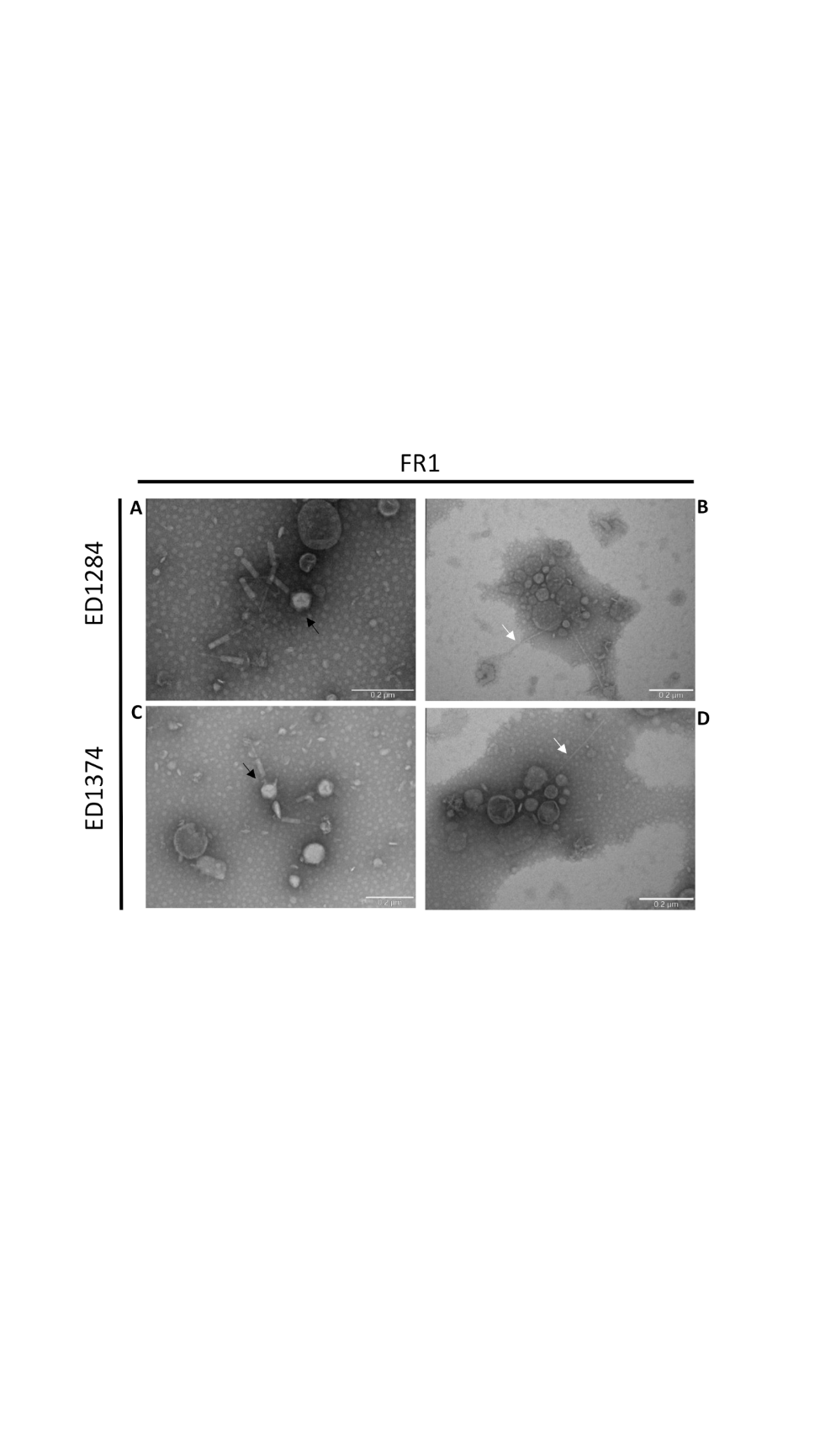
**

**Supplementary Figure 1.** Representative images of TEM (A-B, C-D) micrograph of FR1 obtained from both ED1284 and ED1374 strains showed presence of bacteria pili (white arrows) and phages (black arrows).

**
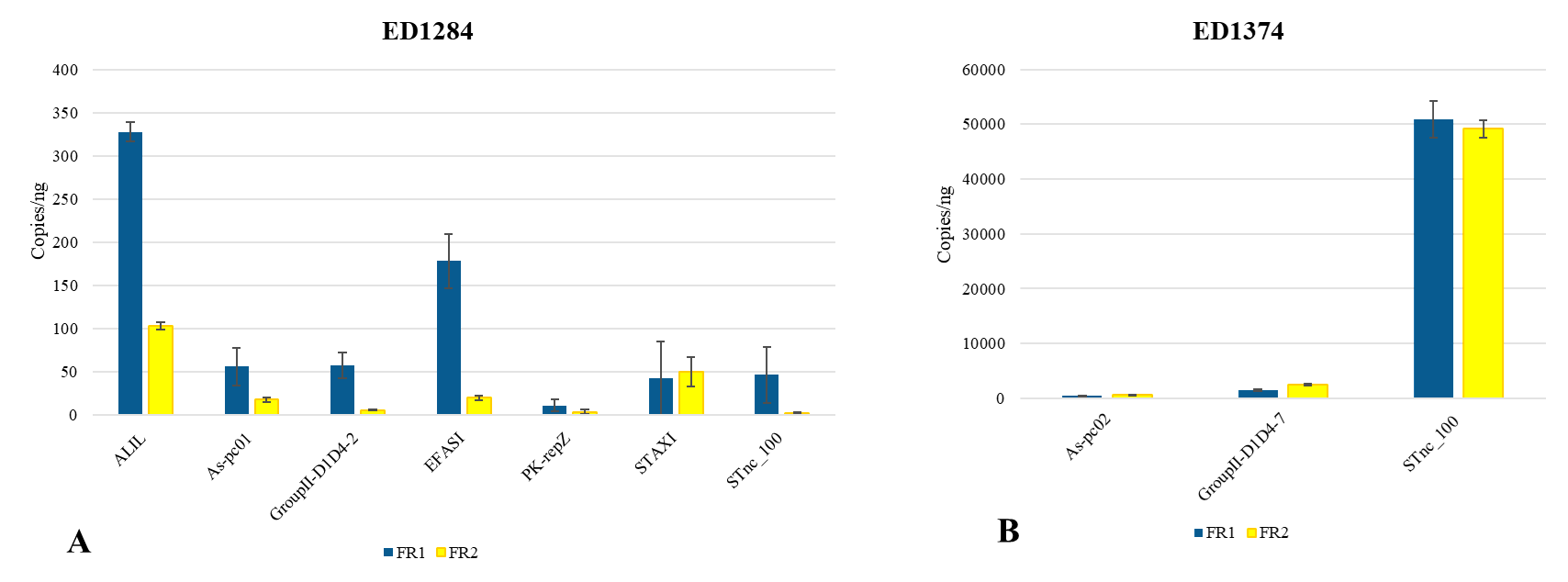
**

**Supplementary Figure 2.** Presence and concentration of small RNAs in FR1 (blue) and FR2 (yellow) of OMVs preparations obtained from ED1284 (A) and ED1374 (B) strains. Results are reported in copies/ng of cDNA.

**
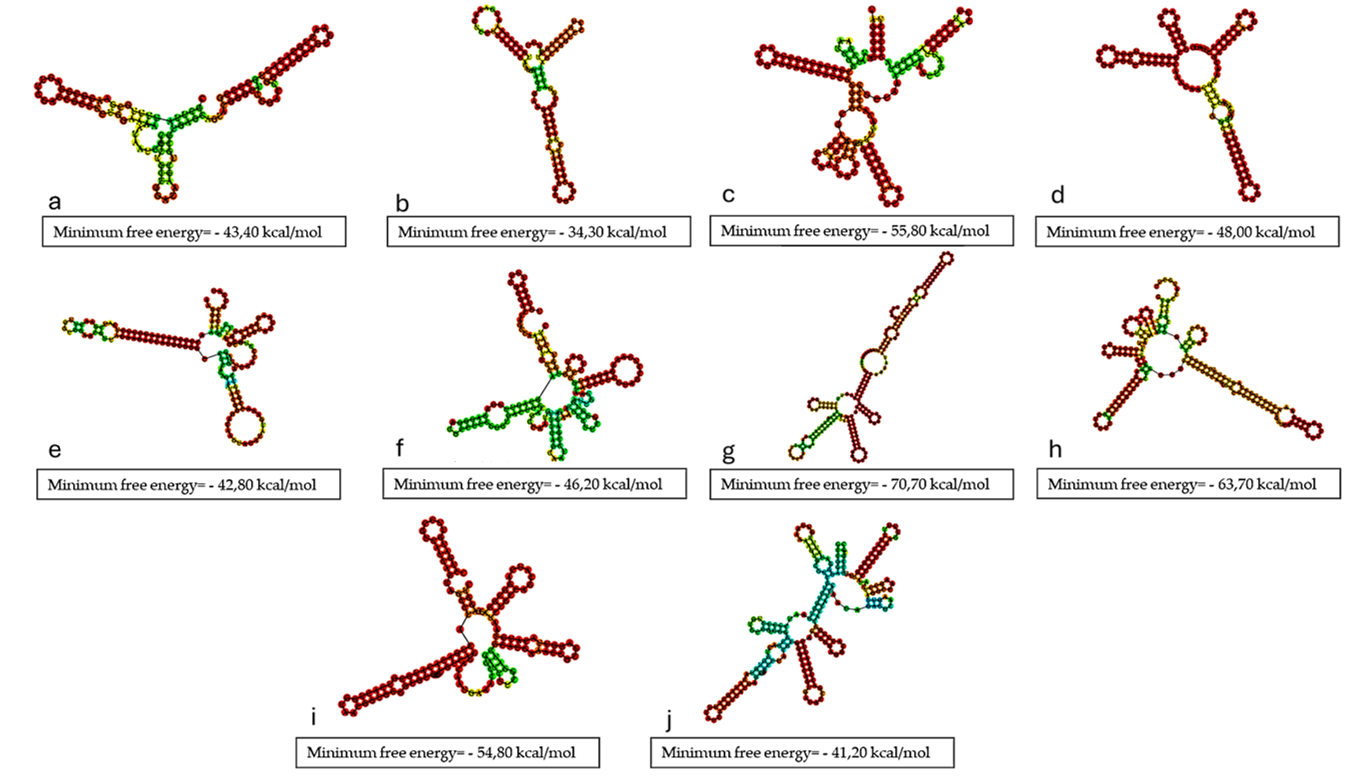
**

**Supplementary Figure 3.** Representation of secondary structures of the identified small RNAs and their minimum free energy (MFE) values obtained by RNAfold. The blue to red colour scale indicates the increased probability for base pairing (blue = 0; red = 1). (a) ALIL, (b) As-pc01, (c) EFASI, (d) GroupII-D1D4-2, (e) PK-repZ, (f) STAXI and (g) STnc_100 from strain ED1284 (O26:H11); (h) As-pc02, (i) GroupII-D1D4-7 and (j) STnc_100 from strain ED1374 (O80:H2).
